# Plethora of Traumatic Lesions of Bilateral Knee Extensor Mechanism in Osteogenesis Imperfecta

**DOI:** 10.3389/fendo.2020.603638

**Published:** 2021-01-21

**Authors:** Peter Kloen, Reggie Charles Hamdy, Niels Hendrik Bech

**Affiliations:** ^1^Department of Orthopedic Surgery, Amsterdam University Medical Center, Amsterdam, Netherlands; ^2^Division of Orthopaedic Surgery, McGill University Health Centre, Shriners Hospital for Children, Montreal, QC, Canada

**Keywords:** patella, fracture, Osteogenesis Imperfecta, avulsion, non-union

## Abstract

**Introduction:**

Injuries to the quadriceps extensor mechanism are rare in patients with Osteogenesis Imperfecta (OI). To the best of our knowledge, non-union of the patella in OI, either as an isolated problem or in combination with an acute fracture, has not been previously reported.

**Case report:**

We describe how we surgically approached both the fracture and the non-union simultaneously. The surgical technique and steps are described in detail. Post-operative course was uneventful and the outcome was favorable, with full return of function for the patient.

**Conclusion:**

A review of various knee extensor mechanism injuries in OI is described as illustrated in a single patient. The unusual simultaneous surgical treatment of a non-union and an acute fracture in the same patella shows that despite the severely compromised bone in this rare bone disease the bone still has a capacity to heal with a functional outcome.

## Introduction

Osteogenesis imperfecta (OI) is an inherited bony dysplasia best known for its fracture susceptibility of the long bones and the spine, deformity, and growth deficiency. Its underlying genetic disorder is a mutation in either one of the collagens I genes (Col 1A1 or Col 1A2) that encode the alpha 1 and alpha 2 chains of collagen. With newly identified mutations since 2006, the original Sillence classification (type I–IV) that was based on clinical presentation, radiographic appearance, and inheritance pattern, has been adapted to now include 21 different OI types (1–5).

The most common OI type is type I. These patients are usually active and ambulatory with a slight shorter stature (6). Most fractures are of the long bones (femur, tibia, and humerus), and spine. Most fractures are sustained at a young age, although it is a life-long problem with an estimated 25% of fractures occurring in adult life. Fractures of smaller bones are relative rare. The increased understanding of the underlying molecular mechanisms has not been mirrored by improvements in treatment (7).

The orthopedic surgeon has an important role in the treatment of these fractures in OI patients, whether operative or non-operative. We describe a patient who sustained traumatic injuries—and post-traumatic complications thereof—to various components of the extensor mechanism of both knees over the course of 15 years. Among these, we report an unusual combination of a fracture and non-union in the same patella. To the best of our knowledge, this combination has not been previously reported. Furthermore, we were unable to find the description of a patellar non-union in OI. In this case report, we present these injuries, their treatment, and outcome, in addition to a review of the literature on extensor mechanism injury of the knee in OI.

## Case Report

A 29-year-old male with a diagnosis of type I osteogenesis imperfecta presented to our emergency room with a painful right knee and inability to extend his knee. He had fallen of a folding chair that collapsed when he tried to sit down. This had caused a forced hyperflexion injury of his right knee.

His past medical history was significant for numerous fractures of both upper and lower extremities. He had sustained bilateral transverse patella fractures 13 years prior to the present injury. These had been treated with suture repair. He also had sustained an avulsion of the inferior pole of the right patella that was treated with suture fixation 3 years prior. He had done reasonably well but then sustained a new injury to this right knee and was diagnosed with a transverse non-union of the patella. It was unclear if this had been in existence since the initial suture fracture repair 13 years prior. He had developed a 20 degrees extension lag of his knee but was still able to do a straight leg raise. Because of the CT finding of the non-union, he was offered surgery but given his minor symptoms and upcoming school exams he declined surgery.

His past medical history also included scoliosis correction T4-L5, operative repair of a left and right (including revision and later hardware removal) olecranon fracture, and a distal humerus fracture. His medication at the time of his presentation included bisphosphonates (risedronate/calcium 35 mg once every week) which he had been taken for years. All his previous orthopedic care had been done at another academic hospital.

**Figure 1:**
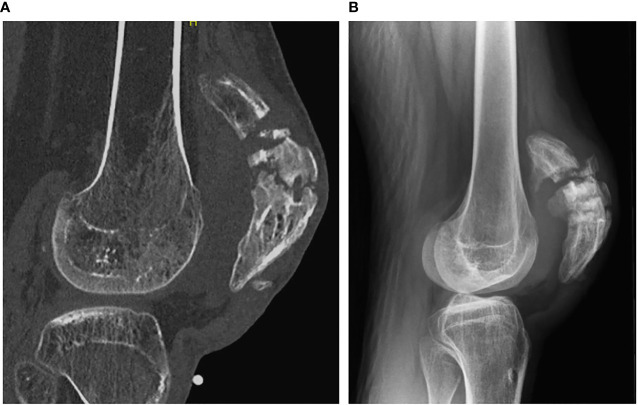
Sagittal CT view **(A)** and lateral radiograph **(B)** of the right patella showing the combination of the acute fracture and longstanding nonunion.

When we first saw him, plain radiographs and a CT of his right knee were obtained (**Figure 1**). These showed a comminuted fresh fracture of the upper half of the right patella proximal to a long-standing transverse non-union. The non-union had sclerotic edges and a gap of approximately 4 mm. Given his inability to extend his knee, we offered surgical repair and, at the same time, an attempt at fixing the long-standing non-union. After discussing the alternative of only fixing the fresh fracture, the patient chose to address surgically both the fracture and non-union with use of homologous bone graft.

Surgery was performed under general anesthesia with the patient in supine position on a radiolucent table. A tourniquet was used. The knee was brought in 20–30 degrees of flexion. The old longitudinal incision of 15 cm was used, extending from the tibia tubercle to 3 finger breadths above the superior pole of the patella. Old suture material was removed. There were no signs on infection. Five deep tissue cultures were taken after which the tourniquet was deflated briefly, and he was given IV antibiotics (Cefazolin 2 gr). A lateral parapatellar arthrotomy was done originating from the tear in the lateral retinaculum. The patella was now inverted to directly visualize the comminuted articular surface. The fracture hematoma was irrigated and debrided. The non-union was identified in the lower half of the patella. There was a clear soft spot but the two parts of the non-union were bridged by non-osseous tissue. The non-union was not opened. With a 1.5 mm drill starting from the fracture side, we made a few drill holes perpendicular through the stiff non-union until blood was noticed to egress from the drill holes. We then first reduced the two parts of the comminuted superior aspect of the patella. This was done using curettes, dental pick, irrigation, and suction and pointed reduction clamps. Perfect alignment was obtained as visualized on the articular surface. The fragments were temporarily transfixed with 1.25 mm K-wires.

A cannulated titanium headless screw (mini Acutrak, Hospital Innovations, Belgium) was then used to fix the two large upper pole fragments. The K-wires were removed. Next this reconstituted superior fragment of the fracture was reduced to the inferior part of the patella including the non-union using two large-pointed reduction clamps and temporary K-wires. From distal two 4.7 mm cannulated headless titanium screws (Acutrak, Hospital Innovations, Belgium) were placed through the non-union from inferior to superior crossing the fracture. Care was taken to bury the screws within the bone as not to irritate the quadriceps or patellar tendon. A figure-of-eight 1 mm steel cerclage wire was then placed through these two 4.7 mm screws, twined, and tightened. The K-wires and reduction clamps were removed. A 2.4/2.7 mm steel Mesh plate (DePuy Synthes, Amersfoort, The Netherlands) was then cut to fit over the dorsal side of the reconstructed patella and fixed with unicortical 2.7 mm locking screws. Each major fragment was fixed by the mesh plate without encroachment of the hardware on the superior and inferior tendinous parts. The superior limb of the plate was placed under the quadriceps, where the inferior part was placed under the patella tendon. Screw placement was observed to be extra-articular through the lateral parapatellar arthrotomy. Two non-absorbable no. 2 sutures were placed through the superior and inferior portion of the plate and then passed through the patellar tendon as a Krackow suture. These sutures were tied with the knee in extension. The retinaculum, subcutaneous tissues, and skin were closed in the usual fashion. The dorsal aspect of the fracture and non-union were covered with 2.5 cc demineralized bone matrix (DBX, DePuy Synthes, Amersfoort, The Netherlands). Total tourniquet time was less than 2 h. The patient was given a hinged knee brace locked in extension for 6 weeks. No active extension was allowed for 6 weeks. After 6 weeks the brace was discontinued, and physical therapy was started. Bisphosphonates were discontinued until there was healing of both the fracture and non-union.

**Figure 2:**
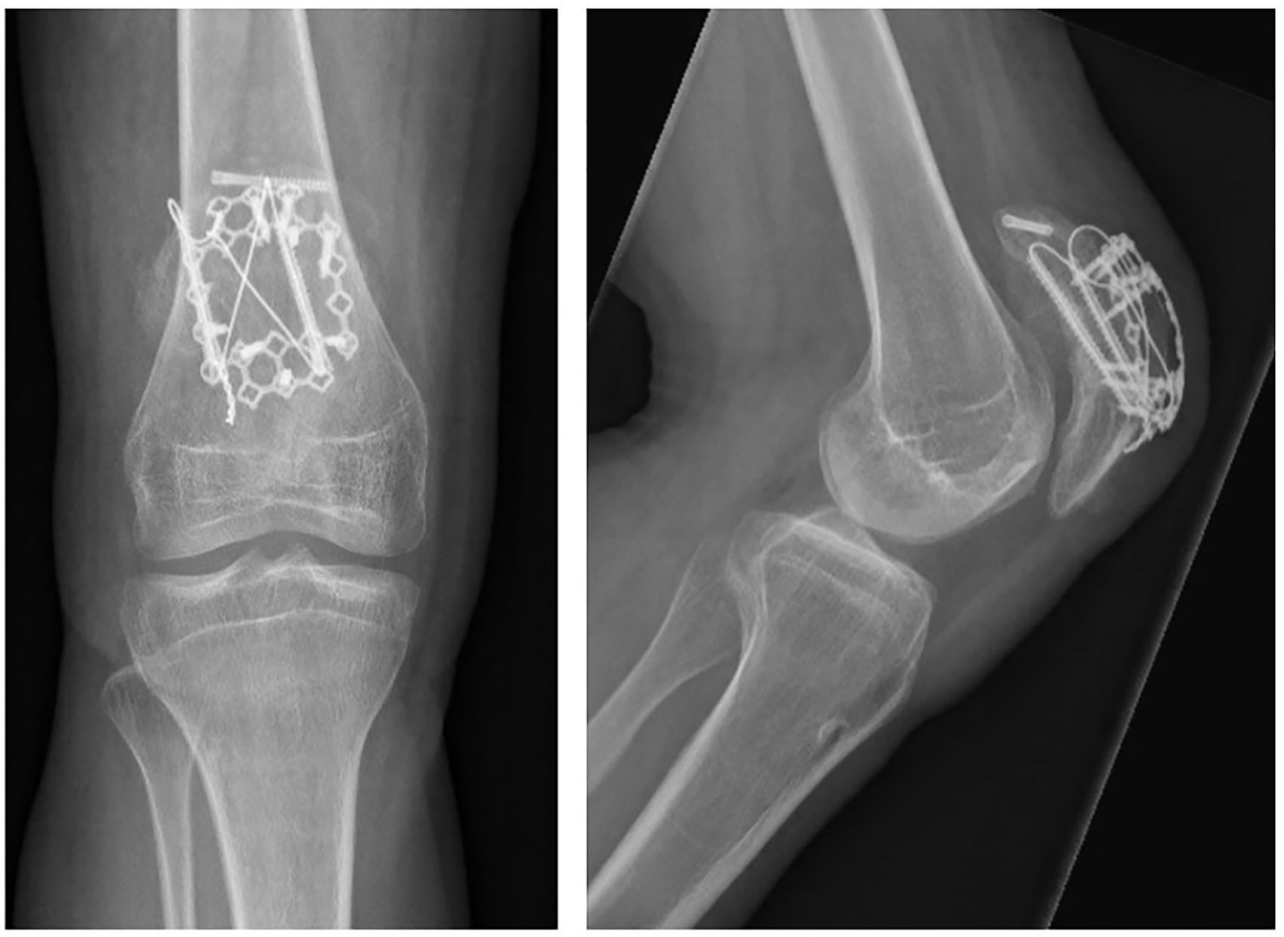
Plain AP and lateral radiographs at 3 months follow-up showing a healed patella.

At 3 months follow up the patient had regained full range of motion of his knee (140-0-0) and reported no pain (**Figure 2**).

**Figure 3:**
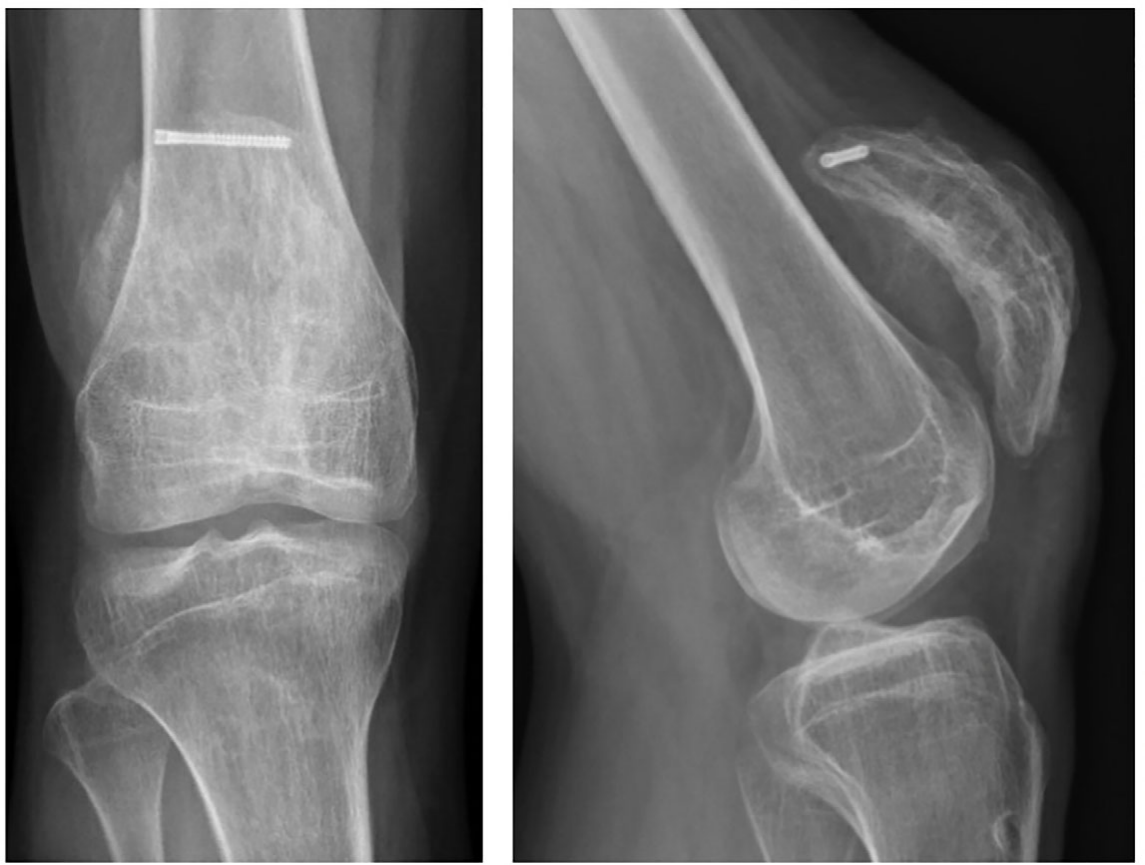
AP and lateral radiographs after hardware removal.

One year after the right knee surgery he returned to his old hospital where the patella hardware on the right was removed as this was bothering him. The patella had solidly healed with an excellent function (**Figure 3**).

**Figure 4:**
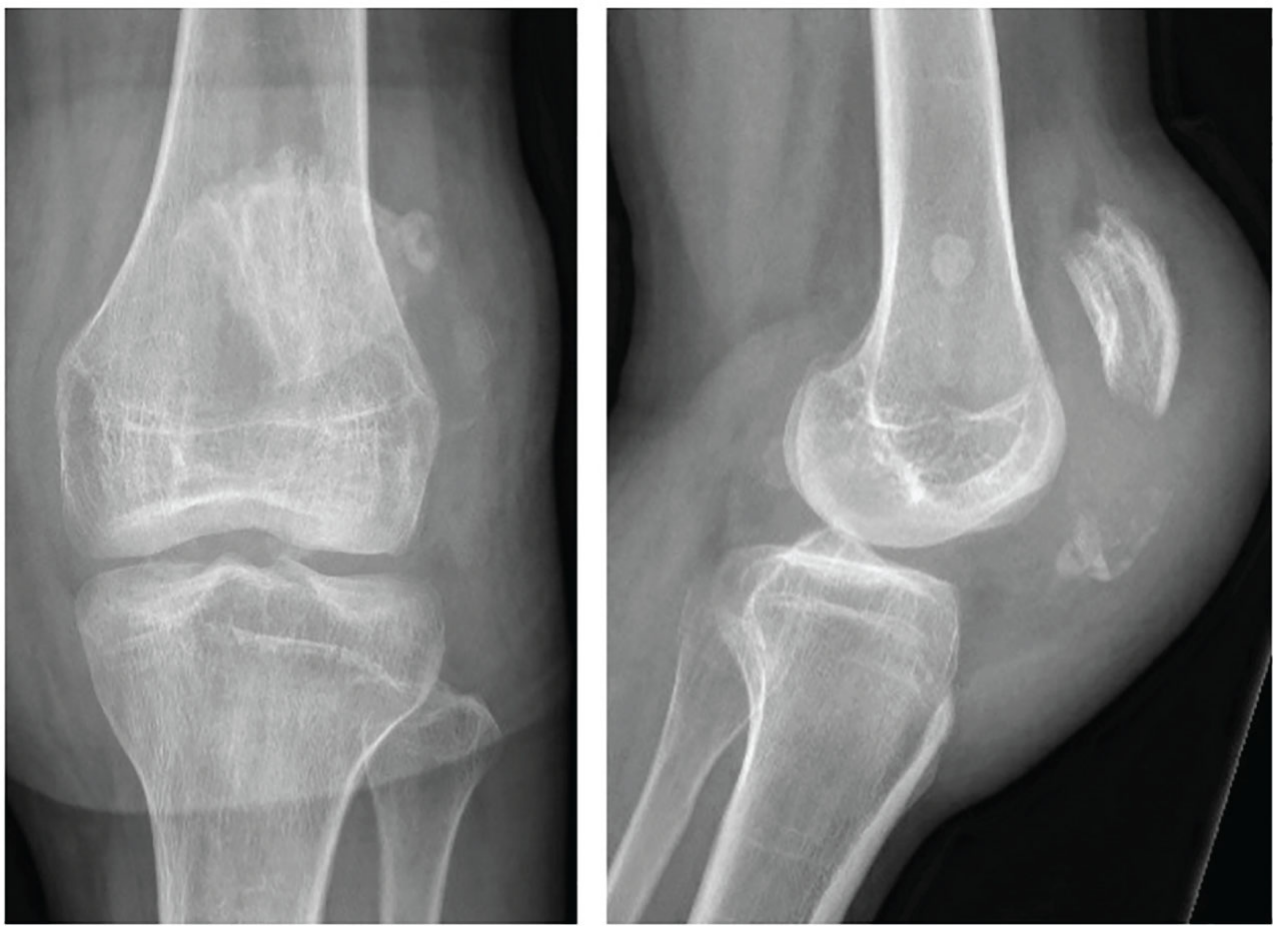
AP and lateral radiographs of the left patella showing an avulsion fracture of the inferior patella pole.

Eight months later the patient was diagnosed with an avulsion fracture of the inferior pole of the left patella (**Figure 4**) for which he underwent suture fixation at an outside hospital. This healed uneventfully within 3 months.

**Figure 5:**
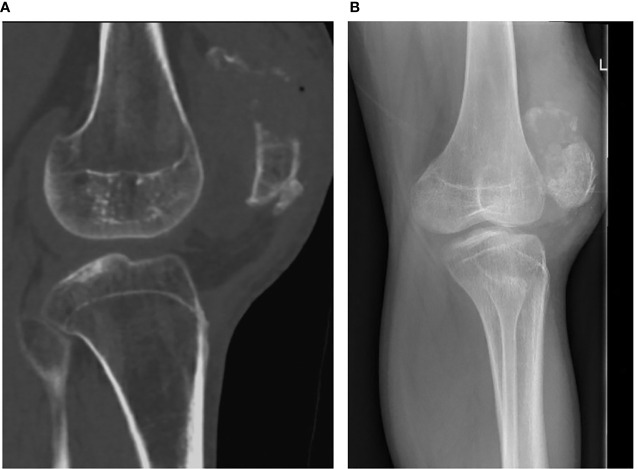
Sagittal CT view **(A)** and lateral radiograph **(B)** of the left patella showing an avulsion fracture of the superior patella pole.

**Figure 6:**
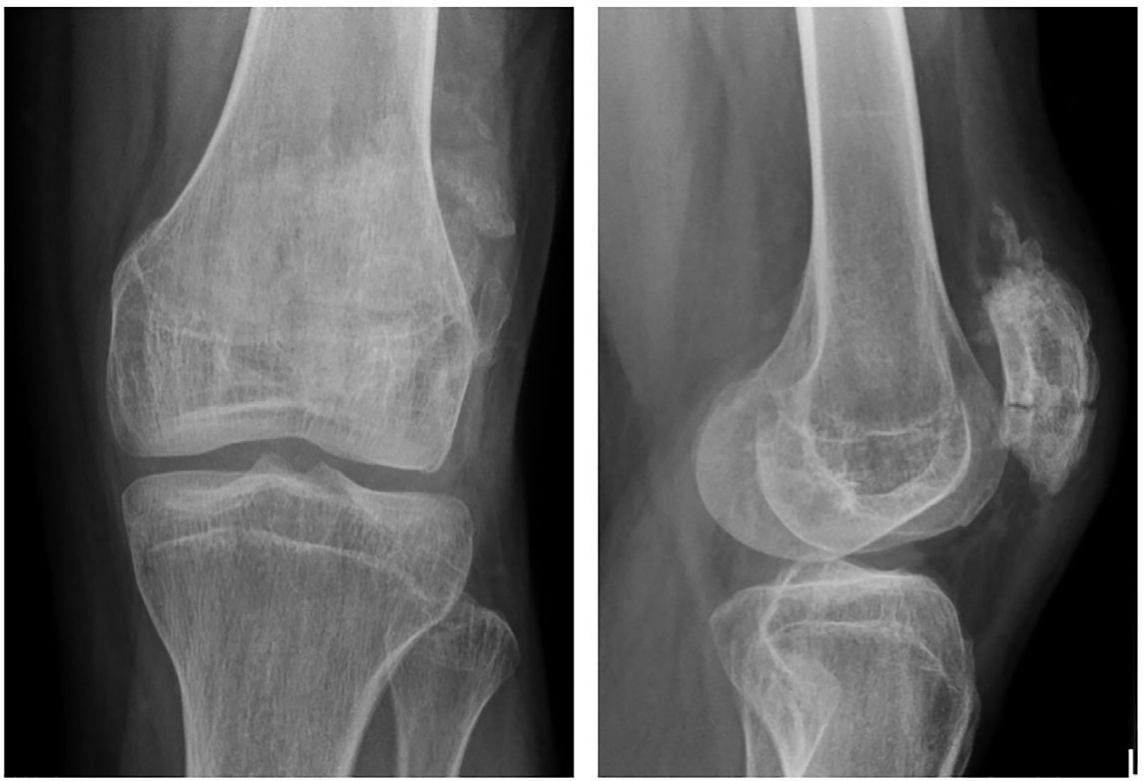
AP and lateral radiographs showing a transverse fracture of the left patella.

**Figure 7:**
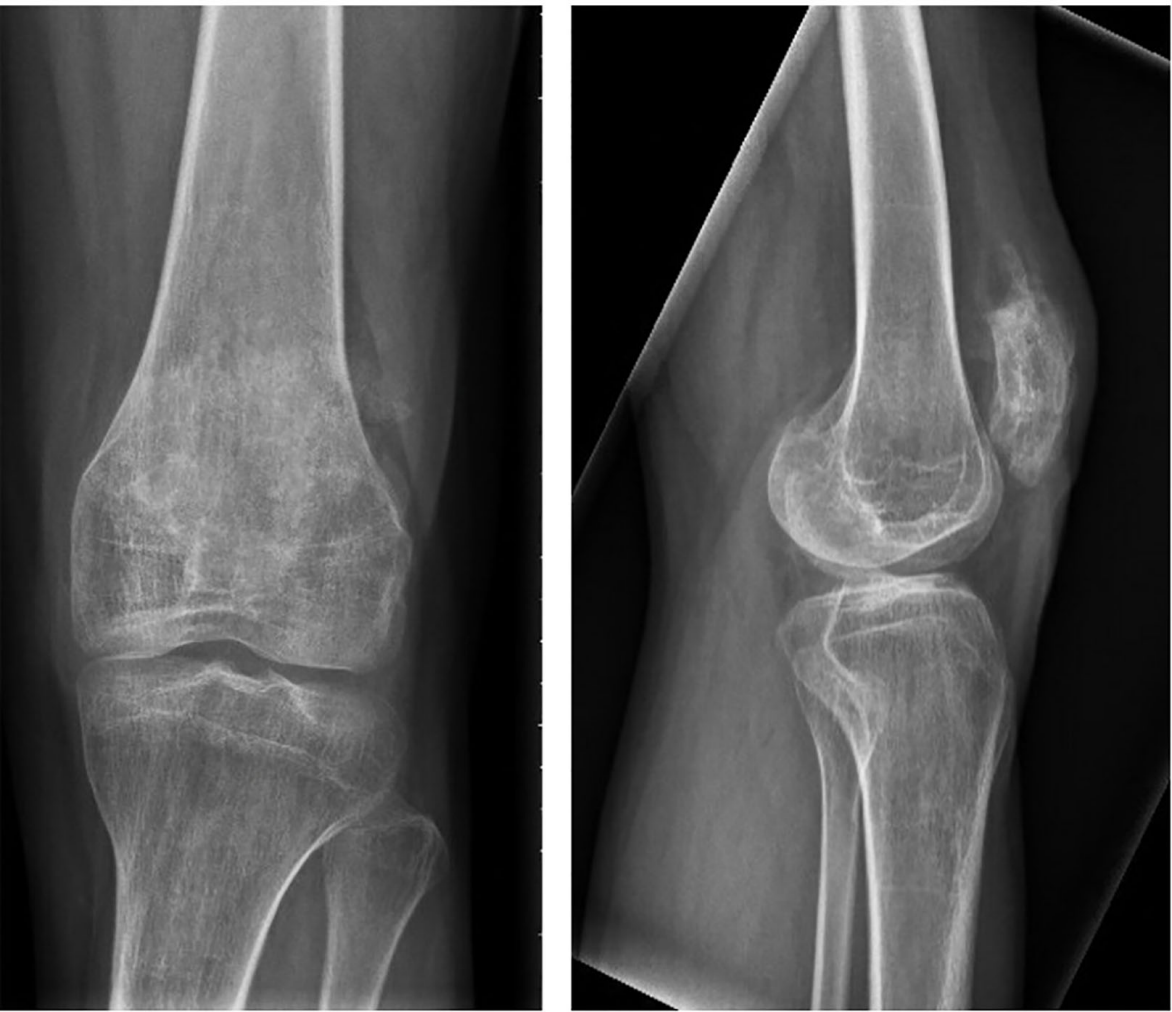
AP and lateral radiographs at 3 months, showing healing of the transverse left patella fracture.

**Figure 8:**
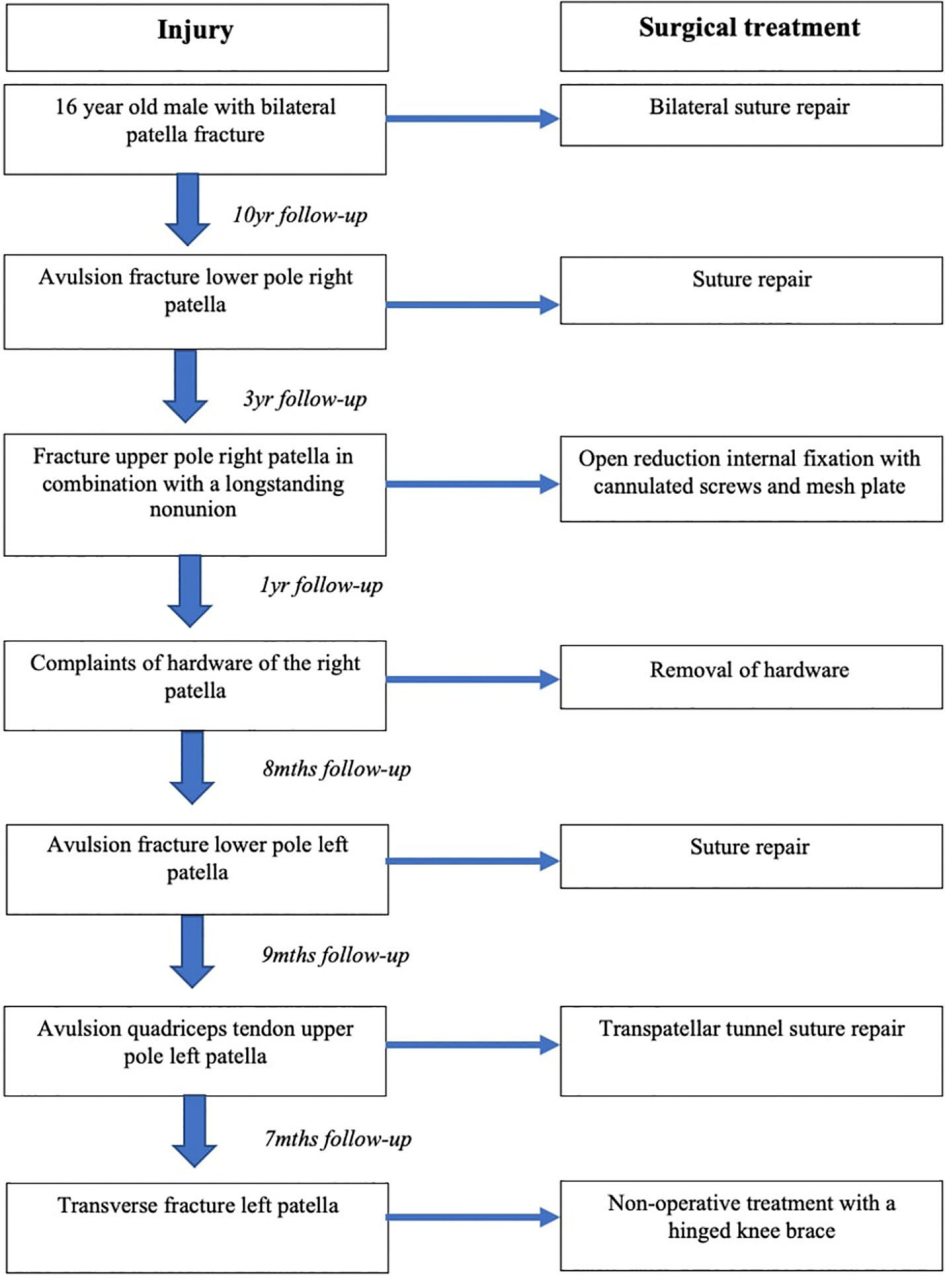
Chronological overview of the patient’s traumatic knee lesions and treatment.

Nine months later he fell off a barstool and sustained an avulsion of the quadriceps tendon of the superior pole of the left patella (**Figure 5**) in addition to a right ankle fracture dislocation. We performed open reduction and internal fixation of his ankle fracture and suture repair through transpatellar tunnels of his left quadriceps’ tendon avulsion. These two injuries recovered uneventfully. Seven months later he again fell off a chair and was diagnosed with a transverse fracture of the left patella (**Figure 6**). As he was still able to do a straight leg raise, we treated him with a hinged knee brace. At 3 months follow up his left patella fracture had healed in anatomic position (**Figure 7**), with a good functional outcome (for a chronologic overview of his traumatic knee lesions see **Figure 8**).

## Overview of the Literature on Extensor Mechanisms Injuries of the Knee in OI

A review was done in accordance with the Prisma statement (www.prisma-statement.org). A comprehensive search was done in PubMed and Embase.com. The search terms (including closely related words and synonyms) included “osteogenesis imperfecta”, “patella”, “knee”, “ligament”, “sleeve”, “fracture”, and “non-union” and “nonunion”. We only included articles in English, there were no date restrictions. Using this strategy, we identified a total of nine suitable articles. After cross checking references three more articles were included. Most of the papers were case reports. The articles were assessed by the first author of this manuscript. A total of 12 were selected for this review (**Table 1**). Of these 12 papers, there were seven that described extensor mechanism injures in OI. They are summarized in **Table 1**. Most of them affected young males with OI type I. Operative treatment was common and with successful outcome in 100% of cases. No complications were listed.

**Table 1:** Overview of the literature on extensor mechanisms injuries of the knee in patients with osteogenesis imperfecta (OI).

Author	Injury	Mechanism of injury	Operative details	Follow-up
ElGuindy et al. (8)	Chronic patellar tendon disruption	Fall downstairs	Reconstruction with allograft bone-tendon-bone	FU 5 yr
	27 yo male, OI type NA	Primary repair		ROM 135-0-5
				IKDC score 58,5%
Mehta and Mahajan (9)	Tibial tubercle avulsion and patellar tendon rupture	Minor tripping and fall	Screw fixation tibial tubercle	FU 7 yr
	11 yo male, OI type NA		Suture (Krakow) and bone anchor	FROM
				10 degrees recurvation
Kim et al. (10)	Simultaneous bilateral patellar tendon rupture	No real trauma	Sutures	FU 10 mo
	55 yo female, Type I OI		Wire loop around patella	ROM 100-0-0
Jansen and Haddad (11)	Distal patellar tendon avulsion	Soccer	Sutures (whipstitch)	FU 1 yr
	29 yo male, Type I OI		Drill holes tubercle and bone anchor	ROM 120-0-0
Kakazu et al. (12)	Avulsion upper pole patella (sleeve fracture)	Spontaneous fracture during walking	Sutures	FU 6 mo
	30 yo male, Type I OI		Quadriceps turn-down	
Wiss et al. (13)	Tibial tubercle avulsion			
	16 yo male, Type I OI	Basketball	Screw fixation	FU 10 mo
	16 yo male, Type I OI	Running	Screw fixation	FU 8 mo
Kothari et al. (14)	Bilateral patellar tendon ruptures	Fall on knee	Suture	FU 7 mo
	27 yo female, Type IB OI		Protection wire	ROM 110-0-0
Khodadadyan-Klostermann et al. (15)	Bilateral tibial tubercle avulsion	Spontaneous fracture during running	Screw fixation	FU 1 yr
	15 yo male, Type I OI			FROM
Figueroa et al. (16)	Spontaneous bilateral quadriceps tendon rupture	Fall	Sutures	FU 4 yr
	28 yo male, Type I OI		Trans patellar tunnels	Lysholm score 95 (excellent)
				FROM
Ogilvie-Harris and Khazim (17)	Avulsion inferior pole	Squash	Sutures	FU 6 wk (normal)
	36 yo male			
	32 yo male	Skiing	Suture anchors	FU 2 yr (normal)
	OI types NA
Salcedo-Duenas et al. (18)	Bilateral quadriceps tendon rupture	Fall on knee	Non-absorbable sutures and transpatellar tunnels	FU 4 yr
	28 yo male, Type I OI			Lysholm score 95 (excellent)
				FROM
Tamborlane et al. (19)	Bilateral tibial tubercle avulsion	Spontaneous fracture during	5mm partially threaded	FU 38 mo
	9 yo male, Type I OI	running	cannulated screw with washer	FROM

FU, Follow up; IKDC, International Knee Documentation Committee; FROM, full range of motion; ROM, range of motion; NA, not available.

## Discussion

Osteogenesis imperfecta (OI) is known for its qualitative or quantitative deficiency of collagen I. As such it can affect not only bone but any other organ or structure containing connective tissue, including tendon, sclerae, ligaments, and skin. Most patients with OI will sustain many fractures especially in childhood. Although many of these fractures can be treated non-operatively, the trend nowadays is to treat long bone fractures operatively with lengthening intra-medullary nails, specifically in the presence of repeated fractures and/or deformities. Intra-articular fractures, however, often warrant surgical treatment, in order to restore the normal anatomy of the articular cartilage.

As previously mentioned, tendons in children with OI may also be weakened by the deficient collagen. Tendon injuries in OI are infrequent and much less common than bony injuries, but have been described affecting the Achilles tendon, triceps tendon, patellar tendon, and quadriceps tendon (8, 14, 16, 17, 20). These later two tendons are part of the extensor mechanism of the knee which also include the patella and the patellar tendon insertion onto the tibial tubercle. Discontinuity of the knee extensor mechanism will prevent knee extension and causes instability during walking. This by itself represents a major disabling injury in any patient, but even more so in an OI patient whose mobility is already compromised by deformity, muscular weakness, and ligamentous laxity. Discontinuity in the extensor mechanism of the knee can be caused by tendon avulsion, tendon rupture, fracture, or combinations thereof. In children there is a fracture subtype known as a sleeve fracture, described in 1997 by Houghton and Ackroyd (21), where a sleeve of cartilage is pulled off the main body of the patella with a bony fragment. This can either be from the inferior pole which is most common, or the superior pole (22). In essence it is a failure of an immature osteochondral junction. It is unclear if a true sleeve fracture can exist in the adult as the ossification of the patella is considered complete in adulthood. Possibly an intrinsic tendon and/or bone problem such as OI can cause a “sleeve fracture” in adulthood (12).

Other causes of a quadriceps or patellar tendon rupture are systemic diseases such as systemic lupus erythematosus (SLE), rheumatoid arthritis, (RA), gout, or hyperparathyroidism, steroid use, or renal disease (23, 24). The underlying pathophysiology of these ruptures varies with the disease process. Avulsions are best treated with sutures via trans-osseous tunnels or bone anchors.

Our patient had already sustained bilateral patellar fractures as a child and also a right patella tendon avulsion fracture as a young adult. Later it was noticed that he had a right patella non-union of unknown age for which treatment was postponed because of school obligations.

When he presented to us for the first time with his fresh comminuted right patella fracture, the co-existing non-union of the patella added another level of complexity to an already challenging problem. The classic definition of a non-union as a fracture that has not healed in 9 months, has recently been modified to a fracture “that will not heal without further invention”. Non-unions in OI occur but have not been addressed in the literature other than in small series or case reports. The popularization of second and third generation oral and intravenous bisphosphonate therapy in OI (25) may increase the risk of a non-union. Biphosphonates inhibit osteoclast activity, potentially having a negative effect on remodeling after fracture healing. This negative side effect of bisphosphonate therapy in OI has been shown after osteotomies but not yet after fractures (26). A recent review concluded that bisphosphonates did not influence fracture healing after wrist, hip or spine fractures (27). The long-term use of bisphosphonates does however seem to be related to atypical femoral fractures (28). Whether biphosphates should be stopped (drug holiday) when an OI patient has an acute fracture is still controversial.

Treatment of the displaced patella fracture is most often done using cerclage wires in comination with K-wires or (cannulated) screws (29). More recently, locked plating for a comminuted patella fracture has become more popular as it provides multiplanar fixation (30).

Treatment of a non-union consists of compression and optimizing of biology in atrophic and oligotrophic non-union (31). To the best of our knowledge, there are no guidelines for simultaneously treating a fracture and a non-union in the same bone. We thought that using a combination of cannulated screws for compression of the non-union and multiplanar plating for buttressing would address both problems in one reconstruction. The use of a lateral parapatellar approach with inversion of the patella would allow an extensive overview of the articular surface. To optimize the biological healing potential of the non-union, the lateral parapatellar approach was also chosen because it had already been shown to not compromise the vascularity (32). Given the compromised bone quality we felt some type of bone graft would be beneficial. The Gold standard for non-union treatment is autologous bone graft. However, given the underlying OI, any bone harvested from the patient would have the same intrinsic problem. Therefore, we chose homologous bone graft in the form of demineralized bone matrix (DBX). Its use has not been specifically studied in OI, but it has shown to be non-inferior in various non-union papers (33).

Delayed healing of osteotomies is often observed in OI patients on bisphosphonates (25). To date it is unclear if bisphosphonate treatment in mild forms of OI can lead to non-union (6). But it seems prudent to withhold bisphosphonates after non-union treatment. Anabolic treatment with teriparatide (parathormone) was also shown to benefit BMD in OI (25). However, in our country, the use of teriparatide for non-union in OI is not current standard of care.

It is important that OI patients have an established relation with orthopedic surgeons that are familiar not only with the disease but who also have experience with non-union treatment. Reports on patella non-unions are scarce. Klasen et al reported on 20 patients with a patella non-union (34). Seven were treated non-operatively (some of whom were minimally symptomatic) whereas the other 13 were operated. Operative management included open reduction and internal fixation (tension band wiring, Bunnell wiring, cerclage wiring, or screw fixation, partial patellectomy, or patellectomy. Only two patients received bone graft but no details on the indications were provided. All operated patients except one healed the non-union. All those treated non-operatively did not heal. Satku and Kumar reported on three patella non-unions that were successfully treated with tension band fixation (35). Uvarai et al. treated 22 neglected patella fractures that had not united with tension band wiring with or without cerclage in 19 and with patellectomy in three (36). Twenty out of 22 had an excellent/good results at year follow up of 5.5 year. Nathan et al published a systematic review on non-union and delayed union of the patella (31). A total of 5 publications was identified, including 45 patients. In 66% of patients, the treatment consisted of tension band wiring. Bone graft was rarely used (2/45). They concluded that tension band wiring is the treatment of choice for patient suitable for reconstruction. Partial of total patellectomy is also an option according to their review (31).

## Conclusion

This case reports describes a variety of consecutive rare traumatic injuries of the extensor mechanism of the knee in a type I OI patient. It is unclear whether suboptimal patellofemoral alignment (patella alta) and/or the deformity of the patella led to increased stress distribution over the tendon-bone juncture and/or the bone. The combination of a fresh fracture proximal to a pre-existing non-union in the same bone is extremely rare. Despite the various injuries and their complication, the patient kept his ambulatory status with a functional range of motion of both knees.

## Author Contributions

PK wrote the first draft of the manuscript and was the treating physician. RCH and NHB contributed to the writing and design. All authors contributed to the article and approved the submitted version.

## Conflict of Interest

The authors declare that the research was conducted in the absence of any commercial or financial relationships that could be construed as a potential conflict of interest.

## References

[1] van Dijk FS Cobben JM Kariminejad A Maugeri A Nikkels PG van Rijn RR Osteogenesis Imperfecta: A Review with Clinical Examples. Mol Syndromol (2011) 2(1):1–20. 10.1159/000332228 22570641 PMC3343766

[2] van Dijk FS Sillence DO Osteogenesis imperfecta: clinical diagnosis, nomenclature and severity assessment. Am J Med Genet A (2014) 164A(6):1470–81. 10.1002/ajmg.a.36545 PMC4314691 24715559

[3] Forlino A Marini JC Osteogenesis imperfecta. Lancet (London England) (2016) 387(10028):1657–71. 10.1016/S0140-6736(15)00728-X PMC7384887 26542481

[4] Forlino A Cabral WA Barnes AM Marini JC New perspectives on osteogenesis imperfecta. Nat Rev Endocrinol (2011) 7(9):540–57. 10.1038/nrendo.2011.81 PMC3443407 21670757

[5] Marini JC Forlino A Bächinger HP Bishop NJ Byers PH de Paepe A Osteogenesis imperfecta. Nat Rev Dis Prim (2017) 3:17052. 10.1038/nrdp.2017.52 28820180

[6] Roberts TT Cepela DJ Uhl RL Lozman J Orthopaedic Considerations for the Adult With Osteogenesis Imperfecta. J Am Acad Orthop Surg (2016) 24(5):298–308. 10.5435/JAAOS-D-15-00275 27100300

[7] Ralston SH Gaston MS Management of Osteogenesis Imperfecta. Front Endocrinol (Lausanne) (2019) 10:924. 10.3389/fendo.2019.00924 32117044 PMC7026366

[8] ElGuindy A Lustig S Servien E Fary C Weppe F Demey G Treatment of chronic disruption of the patellar tendon in Osteogenesis Imperfecta with allograft reconstruction. Knee (2011) 18(2):121–4. 10.1016/j.knee.2010.03.005 20591676

[9] Mehta R Mahajan U Tibial-tubercle avulsion and patellar-tendon rupture in pre-pubertal child with osteogenesis imperfecta(OI): Case report and review of current treatment in OI. J Clin Orthop Trauma (2020) 11(2):339–43. 10.1016/j.jcot.2020.01.013 PMC7026616 32099308

[10] Kim WH Ha SH Lee HJ Simultaneous Bilateral Patellar Tendon Ruptures Associated with Osteogenesis Imperfecta. J Korean Orthop Assoc (2016) 51(5):432–6. 10.4055/jkoa.2016.51.5.432

[11] Jansen JA Haddad FS Distal patellar tendon avulsion fracture in a football player with osteogenesis imperfecta. Knee Surg Sports Traumatol Arthrosc (2012) 20(2):327–30. 10.1007/s00167-011-1595-9 21717214

[12] Kakazu T Tatemoto H Kawamura M Sugita T Sleeve fracture of the upper pole of the patella in an adult with osteogenesis imperfecta. Injury (2003) 34(10):793–4. 10.1016/s0020-1383(02)00201-2 14519364

[13] Wiss DA Schilz JL Zionts L Type III fractures of the tibial tubercle in adolescents. J Orthop Trauma (1991) 5(4):475–9. 10.1097/00005131-199112000-00015 1762011

[14] Kothari P Mohan N Hunter JB Kerslake R Case report. Bilateral simultaneous patellar tendon ruptures associated with osteogenesis imperfecta. Ann R Coll Surg Engl (1998) 80(6):416–8. PMC2503147 10209412

[15] Khodadadyan-Klostermann C Morren R Raschke M Haas N Simultaneous Bilateral Tibial Tubercle Avulsion Fractures in a Boy with Osteogenesis Imperfecta. Eur J Trauma (2003) 29(3):164–7. 10.1007/s00068-003-1203-x

[16] Figueroa D Calvo R Vaisman A Spontaneous and simultaneous bilateral rupture of the quadriceps tendon in a patient with osteogenesis imperfecta: a case report. Knee (2006) 13(2):158–60. 10.1016/j.knee.2005.05.007 16125388

[17] Ogilvie-Harris DJ Khazim R Tendon and ligament injuries in adults with osteogenesis imperfecta. J Bone Joint Surg Br (1995) 77(1):155–6. 10.1302/0301-620X.77B1.7822378 7822378

[18] Salcedo-dueñas JA Castro CT Andrés J Ruptura bilateral de cuadríceps en un paciente con osteogénesis imperfecta. Reporte caso (2009) 23(6):386–9. 20377006

[19] Tamborlane JW Lin DY Denton JR Osteogenesis imperfecta presenting as simultaneous bilateral tibial tubercle avulsion fractures in a child: a case report. J Pediatr Orthop (2004) 24(6):620–2. 10.1097/00004694-200411000-00004 15502558

[20] Dent CM Graham GP Osteogenesis imperfecta and Achilles tendon rupture. Injury (1991) 22(3):239–40. 10.1016/0020-1383(91)90054-i 2071214

[21] Houghton GR Ackroyd CE Sleeve fractures of the patella in children: a report of three cases. J Bone Joint Surg Br (1979) 61-B(2):165–8. 10.1302/0301-620X.61B2.438267 438267

[22] Li Y Yu H Huang B Zhang W Wang Y Liu X Upper pole sleeve fracture of the patella secondary to patellar dislocation: A case report. Med (Baltimore) (2019) 98(24):e16011. 10.1097/MD.0000000000016011 PMC6587639 31192948

[23] Loehr J Welsh RP Spontaneous rupture of the quadriceps tendon and patellar ligament during treatment for chronic renal failure. Can Med Assoc J (1983) 129(3):254–6. PMC1875083 6861063

[24] Bhole R Flynn JC Marbury TC Quadriceps tendon ruptures in uremia. Clin Orthop Relat Res (1985) 195):200–6. 10.1097/00003086-198505000-00023 3978953

[25] Dwan K Phillipi CA Steiner RD Basel D Bisphosphonate therapy for osteogenesis imperfecta. Cochrane Database Syst Rev (2016) 10(10):CD005088. 10.1002/14651858.CD005088.pub4 27760454 PMC6611487

[26] Tauer JT Robinson M-E Rauch F Osteogenesis Imperfecta: New Perspectives From Clinical and Translational Research. JBMR plus (2019) 3(8):e10174. 10.1002/jbm4.10174 31485550 PMC6715783

[27] Shin YH Shin WC Kim JW Effect of Osteoporosis Medication on Fracture Healing: An Evidence Based Review. J Bone Metab (2020) 27(1):15—26. 10.11005/jbm.2020.27.1.15 32190605 PMC7064359

[28] Kates SL Ackert-Bicknell CL How do bisphosphonates affect fracture healing? Injury (2016) 47 Suppl 1(0 1):S65–8. 10.1016/S0020-1383(16)30015-8 PMC4739649 26768295

[29] Sayum Filho J Lenza M Teixeira de Carvalho R Pires OGN Cohen M Belloti JC Interventions for treating fractures of the patella in adults. Cochrane Database Syst Rev (2015) 2):CD009651. 10.1002/14651858.CD009651.pub2 25723760

[30] Lorich DG Fabricant PD Sauro G Lazaro LE Thacher RR Garner MR Superior Outcomes After Operative Fixation of Patella Fractures Using a Novel Plating Technique: A Prospective Cohort Study. J Orthop Trauma (2017) 31(5):241–7. 10.1097/BOT.0000000000000787 28166170

[31] Nathan ST Fisher BE Roberts CS Giannoudis PV The management of nonunion and delayed union of patella fractures: a systematic review of the literature. Int Orthop (2011) 35(6):791–5. 10.1007/s00264-010-1105-6 PMC3103972 20680273

[32] Lazaro LE Cross MB Lorich DG Vascular anatomy of the patella: implications for total knee arthroplasty surgical approaches. Knee (2014) 21(3):655–60. 10.1016/j.knee.2014.03.005 24767718

[33] Hierholzer C Sama D Toro JB Peterson M Helfet DL Plate fixation of ununited humeral shaft fractures: effect of type of bone graft on healing. J Bone Joint Surg Am (2006) 88(7):1442–7. 10.2106/JBJS.E.00332 16818968

[34] Klassen JF Trousdale RT Treatment of delayed and nonunion of the patella. J Orthop Trauma (1997) 11(3):188–94. 10.1097/00005131-199704000-00009 9181502

[35] Satku K Kumar VP Surgical management of non-union of neglected fractures of the patella. Injury (1991) 22(2):108–10. 10.1016/0020-1383(91)90066-n 2037322

[36] Uvaraj NR Mayil Vahanan N Sivaseelam A Mohd Sameer M Basha IM Surgical management of neglected fractures of the patella. Injury (2007) 38(8):979–83. 10.1016/j.injury.2007.02.025 17543968

